# Sexuelle Gesundheitsinformationen in sozialen Medien: Ein systematisches Scoping Review

**DOI:** 10.1007/s00103-021-03431-9

**Published:** 2021-10-14

**Authors:** Nicola Döring, Melisa Conde

**Affiliations:** Institut für Medien und Kommunikationswissenschaft, Technische Universität Ilmenau, Ehrenbergstraße 29, 98693 Ilmenau, Deutschland

**Keywords:** Sexuelle Gesundheit, Gesundheitsinformationen, Sexualaufklärung, Digitale Medien, Forschungssynthese, Sexual health, Health information, Sexual education, Digital media, Research synthesis

## Abstract

**Hintergrund:**

Informationen zur sexuellen und reproduktiven Gesundheit werden zunehmend auch über soziale Medien verbreitet und erreichen dort ein großes Publikum.

**Ziel der Arbeit:**

Vor diesem Hintergrund ist es Ziel des vorliegenden Beitrags, den internationalen Forschungsstand zu sexuellen Gesundheitsinformationen in sozialen Medien erstmals systematisch mit einem Scoping Review aufzuarbeiten. Es sollen 7 Forschungsfragen beantwortet werden, die sich auf den Umfang (F1), die Methoden (F2: Inhaltsanalyse, F3: Qualitätsanalyse) sowie die Ergebnisse (F4: Anbieter, F5: Zielgruppen, F6: Themen, F7: Qualität der Informationen) bisheriger Studien beziehen.

**Material und Methoden:**

Die Untersuchung folgt dem PRISMA-Framework für Scoping Reviews sowie dem Open-Science-Ansatz: Sie ist präregistriert und alle Materialien (Codebuch mit Reliabilitätskoeffizienten) und Daten (Liste der identifizierten Studien, Codierung der Studien) stehen auf dem Server der Open Science Foundation zur Verfügung.

**Ergebnisse:**

Es konnten insgesamt 69 Studien mit 72 Datensätzen identifiziert werden, wobei sich mehr als die Hälfte der Publikationen auf YouTube bezieht (F1). Qualitative und quantitative Methoden der Inhaltsanalyse kommen gleichermaßen zum Einsatz (F2), Qualitätsanalysen sind rar (F3). Bei den Anbietern dominieren Gesundheitslaien (F4). Die Zielgruppen sind meist unspezifiziert (F5). Die in den vorliegenden Studien untersuchten Gesundheitsinformationen in sozialen Medien behandeln ein breites Themenspektrum (F6). Sofern Qualitätseinschätzungen vorgenommen wurden, fielen diese eher negativ aus (F7).

**Diskussion:**

Mehr Forschung ist notwendig, um sexuelle und reproduktive Gesundheitsinformationen in sozialen Medien besser zu verstehen und um ihre Qualität und konstruktive Nutzung zu fördern.

**Zusatzmaterial online:**

Zusätzliche Informationen sind in der Online-Version dieses Artikels (10.1007/s00103-021-03431-9) enthalten.

## Abstract (en)

**Background:**

Information on sexual and reproductive health is increasingly disseminated via social media and reaches a large audience.

**Research aim:**

Against this background, the aim of this paper is to systematically summarize the international state of research on sexual health information on social media for the first time with a scoping review. Seven research questions were explored, relating to the amount (F1), methods (F2: content analysis, F3: quality analysis), and results (F4: providers, F5: target groups, F6: topics, F7: quality of information) of previous studies.

**Materials and methods:**

The study follows the PRISMA framework for scoping reviews as well as the Open Science approach. It is preregistered, and all materials (codebook with reliability coefficients) and data (list of identified studies and coding of studies) are available on the Open Science Foundation server.

**Results:**

A total of 69 studies with 72 data sets were identified, with more than half of the publications referring to YouTube (F1). Qualitative and quantitative methods of content analysis are used equally (F2) and quality analyses are rare (F3). Health laypersons dominate as the information providers (F4). The target groups are mostly unspecified (F5). The health information on social media examined in the previous studies covers a broad range of topics (F6). Where quality assessments were made, they tended to be negative (F7).

**Discussion:**

More research is needed to better understand sexual and reproductive health information on social media and to promote its quality and constructive use.

## Einleitung

Mit *sexuellen Gesundheitsinformationen* sind Informationen gemeint, welche die sexuelle und reproduktive Gesundheit betreffen. Dazu gehören beispielsweise Informationen über die Prävention von und die Intervention bei sexuell übertragbaren Infektionen (STI), sexuellen Übergriffen, ungeplanten Schwangerschaften und sexuellen oder reproduktiven Störungen, aber auch Informationen über sexuelle Identitäten, sexuelle Techniken und Lebensstile sowie sexuelles Vergnügen und Wohlbefinden [1, 2].

Entsprechende Informationen werden im Rahmen formaler sowie informeller Sexualaufklärung vermittelt, etwa durch pädagogische und medizinische Fachkräfte sowie durch Eltern, Peers und Medienpersonen. Entscheidend für das gängige Verständnis von sexuellen und reproduktiven Gesundheitsinformationen ist die Intention der Wissensvermittlung. Daher werden Materialien mit der Hauptintention von Entertainment, Erregung oder Marketing (z. B. erotische Geschichten, Pornovideos, Werbung für Penisverlängerungen) nicht zu den Informationsmaterialien gezählt. Sexuelle Gesundheitsinformationen basieren teils auf Faktenwissen (z. B. wie sicher sind verschiedene Verhütungsmethoden gemäß dem Bewertungsmaßstab Pearl-Index), teils aber auch auf Erfahrungswissen (z. B. wie fühlt man sich als bisexuelles Mädchen und wie kann man sich in der Familie am besten outen). Der Zugang zu umfassenden und evidenzbasierten sexuellen Gesundheitsinformationen wird in Forschung und Praxis als Voraussetzung für sexuelle Gesundheit und als sexuelles Menschenrecht betrachtet [1–4].

In den letzten Jahren ist die Bedeutung *digitaler Medien* für die Verbreitung sexueller Gesundheitsinformationen stark gestiegen. Denn online kann man jederzeit diskret und schamfrei nach sexuellen Informationen suchen, was sowohl jüngere als auch ältere Menschen weltweit immer häufiger tun [5–8]. Gleichzeitig wächst das Angebot an *Online-Sexualaufklärung* beständig [9]. Denn sowohl professionelle Sexualaufklärung als auch sexualbezogene Peer Education werden zunehmend über digitale Medien bereitgestellt, etwa über Websites, Apps, Bots oder Games und natürlich über Social-Media-Plattformen [10–13]. Mit *sozialen Medien* sind Online-Plattformen gemeint, auf denen die Nutzenden sich in eigenen Profilen darstellen, mit anderen vernetzen, Inhalte rezipieren, bewerten und kommentieren sowie eigene Inhalte erstellen und veröffentlichen können [14]. Social-Media-Plattformen erfreuen sich gerade bei den jüngeren Generationen großer Beliebtheit und ermöglichen eine niedrigschwellige Teilnahme an der öffentlichen Online-Kommunikation, das schließt den Austausch sexueller Gesundheitsinformationen ein [15].

So veröffentlichen auf der weltweit meistgenutzten Social-Media-Plattform *YouTube *sowohl Gesundheitsprofis als auch Gesundheitslaien in großer Menge und Vielfalt sexuelle Gesundheitsinformationen. Millionenfach angeschaut und tausendfach kommentiert werden beispielsweise deutschsprachige YouTube-Videos mit Titeln wie: „Daran merkst du, dass du asexuell bist“ (0,8 Mio. Abrufe), „Warum ich die Pille nicht mehr nehme“ (1,5 Mio. Abrufe), „Wie ist das vergewaltigt zu werden“ (2,7 Mio. Abrufe) oder „10 Fakten über Selbstbefriedigung“ (1,8 Mio. Abrufe). Die Bedeutung von Internet und sozialen Medien für die sexuelle Informationsversorgung und damit auch für die sexuelle Gesundheit der Bevölkerung wird weithin anerkannt. Die Bewertung dieser Situation ist jedoch in Forschung und Praxis ambivalent: Denn der *Chance* auf verbesserte sexuelle Informationsversorgung, und damit auch auf verbesserte sexuelle Gesundheit, steht das *Risiko* gegenüber, dass Menschen online unkontrolliert auf verzerrte, lücken- und fehlerhafte Informationen (engl. „misinformation“) sowie auch auf gezielte Falschinformationen (engl. „disinformation“) stoßen und sich ihre sexuelle Gesundheit dadurch verschlechtert [9, 16].

Für eine datenbasierte Einschätzung der sexuellen Gesundheit in Deutschland ist es im Digitalzeitalter somit von großer Bedeutung, nicht nur die selbst berichteten sexualbezogenen Verhaltensweisen, Wissensbestände und Einstellungen der Bevölkerung in Surveys zu ermitteln, sondern auch die sexualbezogenen Online-Informationsangebote und Online-Diskurse systematisch durch Medieninhalts- und Medienqualitätsanalysen zu erfassen und einem regelmäßigen Monitoring zu unterziehen. Dies ist bislang nicht der Fall. Zumindest liegen kaum Studien vor, die untersuchen, welche deutschsprachigen Informationen über sexuelle und reproduktive Gesundheit im Internet öffentlich bereitstehen und welche Qualität diese Angebote der formalen und informellen Online-Sexualaufklärung haben [10]. Der vorliegende Beitrag ruft dazu auf, diese Forschungslücke zu schließen. Dafür wird der internationale Forschungsstand zu Inhalten und Qualität von sexuellen Gesundheitsinformationen in sozialen Medien erstmals im Rahmen eines Scoping Reviews systematisch aufgearbeitet.

## Forschungsstand

Die bisherige Forschung zu sexuellen Gesundheitsinformationen in digitalen Medien gliedert sich in 2 Felder: Studien zu nicht-öffentlichen und zu öffentlichen sexuellen Gesundheitsinformationen.

### Nicht-öffentliche Online-Informationen/-Interventionen

In diesem medizinisch geprägten Forschungsfeld geht es um sexuelle Gesundheitsinformationen und -interventionen, die von Gesundheitsprofis entwickelt und über digitale Medien bereitgestellt werden, um die Zielgruppen besser zu erreichen und/oder die Effektivität der Maßnahmen zu steigern. Es liegen bereits *10 systematische Forschungsreviews* von Evaluationsstudien dieser professionellen Online-Interventionen vor, die z. B. der Prävention von STI oder von ungeplanten Schwangerschaften dienen. Diese Forschungsübersichten weisen zum Teil auf positive Effekte entsprechender Interventionen mittels Websites, mobiler Apps, digitaler Games und sozialer Medien hin [11–13, 16–22]. Der Forschungsfokus liegt dabei auf den Wirkungen der Interventionen im Sinne von Wissenszuwachs sowie Einstellungs- und Verhaltensänderungen, nicht auf der Analyse der bereitgestellten sexuellen Gesundheitsinformationen, da deren Nützlichkeit und inhaltliche Qualität vorausgesetzt werden. Entsprechende Interventionen und die darin enthaltenen sexuellen Gesundheitsinformationen sind in der Regel nicht öffentlich frei zugänglich, sondern werden nur ausgewählten Zielgruppen im Rahmen pädagogischer oder klinischer Projekte bereitgestellt.

### Öffentliche Online-Informationen

In diesem interdisziplinären Forschungsfeld geht es um sexuelle Gesundheitsinformationen, die von Gesundheitsprofis und/oder von Gesundheitslaien entwickelt und über das Internet öffentlich verbreitet werden. Dabei kann es sich um Websites [23] oder um Inhalte auf Social-Media-Plattformen wie Facebook [24], YouTube [25], Instagram [26] oder Twitter [27] handeln. Entsprechende Inhalte sind in der Regel kostenfrei und niedrigschwellig zugänglich. Der Forschungsfokus liegt hier auf der Analyse der Inhalte und der Qualität der bereitgestellten sexuellen Gesundheitsinformationen, nicht auf deren Wirkungen. Die bisherige Forschung zu öffentlich verfügbaren sexuellen Gesundheitsinformationen in sozialen Medien wurde bislang noch nicht in einem Forschungsreview zusammengefasst.

## Forschungsziel

Vor diesem Hintergrund ist es Ziel des vorliegenden Beitrags, den internationalen Forschungsstand zu öffentlich frei verfügbaren, sexuellen Gesundheitsinformationen in sozialen Medien systematisch mit einem Scoping Review herauszuarbeiten.

Da die Social-Media-Landschaft vielfältig und dynamisch ist, wird die Analyse auf die 8 in Deutschland bei Jugendlichen und Erwachsenen meistgenutzten Social-Media-Plattformen fokussiert [28, 29]: 1. YouTube, 2. Facebook, 3. Instagram, 4. Snapchat, 5. Twitter, 6. TikTok, 7. Twitch und 8. Pinterest.

Das vorliegende Forschungsreview dient der Beantwortung von 7 Forschungsfragen (F1–F7). Wie bei jedem Scoping Review interessiert zunächst die Menge der vorliegenden Studien:

### F1.

Wie viele Studien liegen zu sexuellen Gesundheitsinformationen auf den 8 ausgewählten Social-Media-Plattformen vor?

Weiterhin interessiert das methodische Vorgehen dieser Studien:

### F2.

Welche Methoden zur Analyse der Inhalte der sexuellen Gesundheitsinformationen in sozialen Medien werden bislang in der Forschung eingesetzt?

### F3.

Welche Methoden zur Bewertung der Qualität der sexuellen Gesundheitsinformationen in sozialen Medien werden bislang in der Forschung verwendet?

Schließlich interessieren die Befunde der vorliegenden Studien:

### F4.

Von wem stammen die untersuchten sexuellen Gesundheitsinformationen in sozialen Medien?

### F5.

Welche Zielgruppen haben die untersuchten sexuellen Gesundheitsinformationen in sozialen Medien?

### F6.

Welche Themen der sexuellen und reproduktiven Gesundheit behandeln die untersuchten sexuellen Gesundheitsinformationen in sozialen Medien und welche Wissensform wird dabei bereitgestellt?

### F7.

Welche Qualität haben die untersuchten sexuellen Gesundheitsinformationen in sozialen Medien?

## Methode

Bei der vorliegenden Forschungssynthese handelt es sich um ein *Scoping Review* [30]. Das Scoping Review ist darauf ausgerichtet, den aktuellen Stand in einem heterogenen Forschungsfeld zu erarbeiten und dabei sowohl Methoden als auch Ergebnisse bisheriger Forschung darzustellen. Bei einem Scoping Review wird die eingeschlossene Literatur nicht nur quantifiziert (z. B. in Tabellen), sondern die Ergebnisse werden auch deskriptiv narrativ zusammengefasst [31]. Die Darstellung von Methodik und Ergebnissen der vorliegenden Forschungsübersicht folgt dem PRISMA-Framework (PRISMA = Preferred Reporting Items for Systematic Reviews and Meta-Analyses) für Scoping Reviews (PRISMA-ScR; [32]). Im Sinne des *Open-Science-Ansatzes *ist die vorliegende Studie präregistriert und alle Daten und Materialien sind in englischer Sprache auf dem Server der Open Science Foundation (https://osf.io/c8jbx/) sowie teilweise auch als Onlinematerial zu diesem Beitrag hinterlegt.

### Einschlusskriterien für relevante Literatur

Einbezogen wurden alle Studien, die folgende Inklusionskriterien erfüllen:

1. Studie behandelt sexuelle Gesundheitsinformationen auf einer oder mehreren der 8 ausgewählten Social-Media-Plattformen (1. YouTube, 2. Facebook, 3. Instagram, 4. Snapchat, 5. Twitter, 6. TikTok, 7. Twitch und/oder 8. Pinterest),
2. Studie untersucht die Inhalte und/oder die Qualität dieser Gesundheitsinformationen,
3. Studie ist empirisch ausgerichtet, d. h., es werden Daten ausgewertet, deren Erhebung und Analyse methodisch nachvollziehbar dargestellt sind,
4. Publikation in deutscher oder englischer Sprache und
5. Publikation mit wissenschaftlicher Qualitätssicherung (Peer-Review-Verfahren).

### Suche nach relevanter Literatur

Zur Literatursuche wurden die 3 wissenschaftlichen Literaturdatenbanken (1) MEDLINE/PubMed, (2) PsycINFO und (3) Scopus genutzt. Als Suchbegriffskombination wurde „sexual“/„sexuell“ jeweils mit dem Namen der Social-Media-Plattform kombiniert. Diese breite Suchstrategie wurde gewählt, um alle Studien mit Sexualitätsbezug einzubeziehen. Die Suchsyntax ist im Onlinematerial zu finden. Die Datenbankrecherche wurde ergänzt durch eine manuelle Recherche in der eher unstrukturierten, aber sehr inklusiven Literaturdatenbank Google Scholar sowie in den Literaturverzeichnissen der aufgefundenen Studien.

### Auswahl der relevanten Literatur

**Figure 1:**
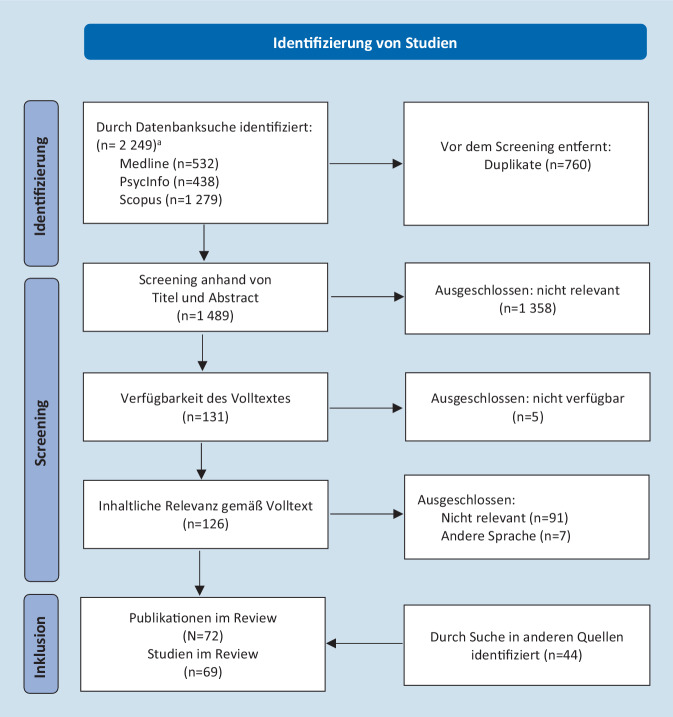

Die durch die Suche identifizierten Quellen durchliefen einen mehrstufigen Screeningprozess. Dabei wurden zunächst alle Duplikate entfernt. Dann wurden die Quellen anhand ihres Titels und Abstracts hinsichtlich der Einschlusskriterien selektiert. Anschließend wurden für die verbliebenen Publikationen die Volltexte beschafft und wiederum hinsichtlich der Einschlusskriterien geprüft. Auf diese Weise wurden schließlich 69 Studien in das Scoping Review eingeschlossen. 3 dieser Studien beinhalten jeweils Analysen zu 2 Social-Media-Plattformen, sodass insgesamt 72 analysierte Datensätze und somit *N* = 72 Publikationen eingegangen sind. Der Auswahlprozess ist in Abb. 1 dargestellt. Alle eingeschlossenen Publikationen sind in Tab. 1 zu finden. Die eingeschlossenen Studien wurden zwischen 2008 und 2021 publiziert, stammen überwiegend aus dem englischsprachigen Raum (USA, Kanada, Vereinigtes Königreich, Australien) und haben vielfältige disziplinäre Hintergründe (v. a. Kommunikations‑, Medien‑, Sozial- und Gesundheitswissenschaften sowie Medizin). 4 Publikationen beziehen sich auf deutschsprachige sexuelle Gesundheitsinformationen [33–36].

**Table 1:** 

Nr.	Autor:innen (Jahr)	Social-Media-Plattform (F1)	Inhaltsanalytische Methode (F2)	Qualitätsanalyse (F3)	Anbieter^a^ (F4)	Zielgruppe genannt (F5)	Themen Anzahl^b^ (F6)	Qualitätsausprägung^c^ (F7)
1	Ache und Wallace (2008; [37])	YouTube	Quantitativ manuell	–	Profis Laien Sonstige	–	1	–
2	Adams-Santos (2020; [38])	YouTube	Qualitativ	–	Laien	+	1	–
3	Afnan et al. (2019; [39])	Instagram	Quantitativ manuell	–	Laien	–	1	–
4	Alaggia und Wang (2020; [40])	Twitter	Qualitativ	–	Laien	–	1	–
5	An et al. (2014; [41])	Twitter	Quantitativ computational	–	Laien	–	1	–
6	Balasubramanian et al. (2020; [42])	Twitter	Quantitativ manuell	–	Profis Laien	–	1	–
7	Basch und MacLean (2019; [26])	Instagram	Quantitativ manuell	–	–	–	1	–
8	Berriche und Altay (2020; [43])	Facebook	Quantitativ manuell	–	Sonstige	–	2	–
9	Blakemore et al. (2020; [44])	Instagram	Quantitativ manuell	–	Profis Laien	–	1	–
10	Blakemore et al. (2020) [44]	Twitter	Quantitativ manuell	–	Profis Laien	–	1	–
11	Bogen et al. (2019a; [45])	Twitter	Qualitativ	–	Laien	–	1	–
12	Bogen et al. (2019b; [46])	Twitter	Qualitativ	–	Laien	–	1	–
13	Bogen et al. (2020; [27])	Twitter	Qualitativ	–	Laien	+	1	–
14	Breen et al. (2016; [47])	Twitter	Quantitativ computational	–	–	–	1	–
15	Briones et al. (2012; [48])	YouTube	Quantitativ manuell	–	Profis Laien Sonstige	–	1	–
16	Chowdhury und Fileborn (2020; [49])	Facebook	Qualitativ	–	Laien	+	1	–
17	Cornelius et al. (2019; [50])	Twitter	Quantitativ manuell	–	Profis Laien Sonstige	–	4	–
18	Culha et al. (2021; [51])	YouTube	Quantitativ manuell	+	Profis Laien Sonstige	+	1	Mittel
19	Cunningham (2014; [52])	YouTube	Qualitativ	–	Laien	–	2	–
20	Deal et al. (2020; [53])	Twitter	Quantitativ manuell	–	Laien	–	1	–
21	Del Casino Jr und Brooks (2015; [54])	YouTube	Qualitativ	–	Profis Laien Sonstige	–	3	–
22	Döring (2018; [34])	YouTube	Quantitativ manuell	–	Profis Laien Sonstige	+	6	–
23	Döring (2017; [33])	YouTube	Quantitativ manuell	–	Profis Laien Sonstige	–	1	–
24	Döring et al. (2019; [35])	YouTube	Qualitativ	–	Profis Laien Sonstige	–	1	–
25	Döring et al. (2019; [35])	Twitter	Qualitativ	–	Profis Laien	–	1	–
26	Ekram et al. (2019; [55])	YouTube	Quantitativ manuell	+	–	–	1	Gering
27	Endriani et al. (2020; [56])	YouTube	Qualitativ	–	Laien	+	2	–
28	Fode et al. (2020; [57])	YouTube	Quantitativ manuell	+	Profis Laien	+	1	Gering
29	Gabarron et al. (2014; [58])	Twitter	Quantitativ manuell	–	Profis Laien Sonstige	–	1	–
30	Garcia und Vemuri (2017; [59])	YouTube	Quantitativ manuell	–	Laien Sonstige	+	4	–
31	García Jiménez und Montes Vozmediano (2020; [60])	YouTube	Qualitativ	–	Laien	–	1	–
32	Green et al. (2015; [61])	YouTube	Qualitativ	–	Laien	–	1	–
33	Guidry et al. (2020; [62])	Instagram	Quantitativ manuell	–	Profis Laien Sonstige	+	1	–
34	Guidry et al. (2020; [62])	Twitter	Quantitativ manuell	–	Profis Laien Sonstige	+	1	–
35	Gul und Diri (2019; [25])	YouTube	Quantitativ manuell	+	Profis Laien Sonstige	+	1	Mittel
36	Hansen et al. (2016; [63])	YouTube	Quantitativ manuell	+	Profis Laien Sonstige	–	1	–
37	Harlow et al. (2018; [64])	Twitter	Qualitativ	–	Laien	+	1	–
38	Hosterman et al. (2018; [65])	Twitter	Qualitativ	–	Profis Laien Sonstige	+	1	–
39	Johnston (2017; [66])	YouTube	Qualitativ	–	Profis Laien	–	6	–
40	Kecojevic et al. (2018; [67])	YouTube	Quantitativ manuell	–	Profis Laien Sonstige	+	1	–
41	Keller et al. (2018; [68])	Twitter	Qualitativ	–	Laien	+	1	–
42	Kelly-Hedrick et al. (2018; [69])	YouTube	Quantitativ manuell	–	Profis Laien Sonstige	–	2	–
43	Khawaja et al. (2017; [70])	Facebook	Quantitativ manuell	+	Profis Sonstige	+	6	Gering
44	Ku et al. (2020; [71])	YouTube	Quantitativ manuell	+	Profis Laien	+	2	Gering
45	LeBeau et al. (2020; [72])	Snapchat	Qualitativ	–	Sonstige	+	5	–
46	Levinson et al. (2020; [73])	YouTube	Qualitativ	–	Laien	+	2	–
47	Lohmann et al. (2018; [74])	Twitter	Quantitativ manuell	–	Profis Laien Sonstige	–	4	–
48	Lovelock (2017; [75])	YouTube	Qualitativ	–	Laien	+	1	–
49	Lovelock (2019; [76])	YouTube	Qualitativ	–	Laien	+	1	–
50	Mack (2016; [77])	YouTube	Qualitativ	–	Laien	–	2	–
51	Martini (2020; [36])	Twitter	Quantitativ manuell	–	Laien Sonstige	+	1	–
52	Martino et al. (2021; [78])	YouTube	Qualitativ	–	Laien	+	1	–
53	Massey et al. (2016; [79])	Twitter	Quantitativ computational	–	–	–	1	–
54	McBean (2014; [80])	YouTube	Qualitativ	–	Laien	+	1	–
55	McLaughlin et al. (2016; [81])	Twitter	Quantitativ manuell	–	Profis Laien Sonstige	–	1	–
56	Miller (2017; [82])	YouTube	Quantitativ manuell	–	Laien	+	1	–
57	Miller (2019; [83])	YouTube	Qualitativ	–	Laien	+	1	–
58	Morris und Anderson (2015; [84])	YouTube	Qualitativ	–	Laien	+	1	–
59	Nguyen und Allen (2018; [85])	YouTube	Quantitativ manuell	–	Laien	+	1	–
60	Nobles et al. (2020; [86])	Instagram	Quantitativ computational	–	–	–	2	–
61	Onanuga (2020; [87])	Twitter	Qualitativ	–	Laien	–	1	–
62	Paul et al. (2017; [88])	YouTube	Quantitativ manuell	+	Profis Laien Sonstige	–	1	Gering
63	Perez-Torres et al. (2018; [89])	YouTube	Qualitativ	–	Laien	+	1	–
64	Raun (2015; [90])	YouTube	Qualitativ	–	Laien	–	1	–
65	Riggs und Bartholomaeus (2018; [91])	YouTube	Qualitativ	–	Laien	+	1	–
66	Schwartz und Grimm (2017; [92])	Twitter	Quantitativ manuell	–	Profis Laien Sonstige	–	1	–
67	Stephen und Cumming (2012; [93])	YouTube	Quantitativ manuell	–	–	+	3	–
68	Syred et al. (2014; [94])	Facebook	Qualitativ	–	Laien	+	1	–
69	Tortajada et al. (2020; [95])	YouTube	Qualitativ	–	Laien	+	1	–
70	Warren et al. (2021; [96])	YouTube	Quantitativ manuell	+	Profis Laien	+	1	Gering
71	Whiteley et al. (2020; [97])	YouTube	Quantitativ manuell	+	Profis	–	1	–
72	Yeo und Chu (2017; [24])	Facebook	Quantitativ manuell	–	Laien	–	5	–

^a^Gliederung der Anbieter in 3 Gruppen: Profis (Fachleute für sexuelle Gesundheit, Organisationen der sexuellen Gesundheit), Laien (Laien/Peer Sex Educators), Sonstige (Medienorganisationen/Fachleute für Medien und Kommunikation, kommerzielle Anbieter, andere Anbieter)

^b^Anzahl der behandelten Themenbereiche aus 6 Themenbereichen (1 HIV/STI, 2 geschlechtliche und sexuelle Identitäten, 3 sexuelle Gewalt, 4 Fruchtbarkeit und Verhütung, 5 sexuelle und reproduktive Störungen, 6 sexuelle Funktionen, Lebensstile und Vergnügen)

^c^Klassifikation der Qualitätsausprägung als gering (51–100 % – also mehr als die Hälfte – der Untersuchungseinheiten mit Qualitätsmängeln), mittel (11–50 %), hoch (0–10 %; Tab. 4)

### Auswertung der relevanten Literatur

Zur Auswertung der eingeschlossenen Studien wurde ein selbst entwickeltes Codebuch genutzt, das die zur Beantwortung der 7 Forschungsfragen notwendigen 29 Kategorien enthält (siehe Onlinematerial). Das Codebuch wurde in einem Pretest auf Reliabilität geprüft. Dazu wurden 38 zufällig ausgewählte Publikationen aus dem Gesamtpool der Publikationen unabhängig voneinander von 2 geschulten Personen codiert und die Inter-Coder-Übereinstimmungen statistisch ermittelt. Es zeigte sich, dass alle Kategorien des Codebuchs sehr gute Reliabilitäten aufwiesen (Cohens Kappa von 0,65–1,0; Durchschnittswert: 0,88 und prozentuale Übereinstimmung von 87–100 %, Durchschnittswert: 96 %; die vollständigen Ergebnisse des Reliabilitätstests sind im Onlinematerial zu finden). Die Codierung aller Studien erfolgte dann durch eine geschulte Codiererin. Eine Übersicht aller 72 eingeschlossenen Studien findet sich in Tab. 1. Der resultierende Datensatz ist zudem auf https://osf.io/c8jbx/ abgelegt. Die Ergebnisdarstellung erfolgt anhand textueller und tabellarischer Darstellungen der absoluten bzw. relativen Häufigkeiten der codierten Studienmerkmale. Zudem werden die Merkmale und Befunde der Studien narrativ beschrieben.

## Ergebnisse

### Umfang der bisherigen Forschung

Durch das Scoping Review wurden 69 Studien mit 72 analysierten Datensätzen identifiziert, die sich empirisch mit den Inhalten und der Qualität von öffentlich frei zugänglichen sexuellen Gesundheitsinformationen in sozialen Medien befassen. Die Mehrzahl der *N* = 72 Publikationen, nämlich 39 von 72 (54,2 %), beschäftigt sich mit YouTube, gefolgt von Twitter (30,6 %). Jeweils 5 Studien befassen sich mit Facebook und mit Instagram, eine mit Snapchat und keine einzige mit TikTok, Twitch oder Pinterest (F1).

### Methoden der bisherigen Forschung

#### Methoden der Inhaltsanalyse

Zur empirischen Analyse der Inhalte von sexuellen Gesundheitsinformationen in sozialen Medien werden in der bisherigen Forschung sowohl qualitative (44,4 % der Publikationen) als auch quantitative (55,6 %) Methoden der Medieninhaltsanalyse eingesetzt, wobei die quantitativen Methoden sich noch in die beiden Untergruppen manuelle Analyse (50 %) und computationale (rechnergestützte) Analyse (5,6 %) unterteilen lassen (F2).

Qualitative Inhaltsanalysen untersuchen meist Stichproben im ein- bis unteren zweistelligen Bereich. So wurden in einer qualitativen Studie beispielsweise *N* = 25 englischsprachige YouTube-Videos zum Thema Viagra nach theoretischen Überlegungen bewusst ausgewählt und mittels *Grounded Theory Approach* interpretativ hinsichtlich ihrer Botschaften zu „Pharmasexualität“ ausgewertet [54].

Bei den quantitativ manuellen Analysen codieren Menschen das Social-Media-Material mithilfe eines zuvor festgelegten *Codebuchs*. Typisch sind dabei Materialstichproben im oberen zwei- bis dreistelligen Bereich. Eine solche quantitativ manuelle Analyse wollte herausarbeiten, wie der medikamentöse HIV-Schutz mittels oraler HIV-Präexpositionsprophylaxe (HIV-PreP) auf YouTube dargestellt wird [67], denn die 2012 in den USA und 2016 in Deutschland zugelassene orale HIV-PrEP („Anti-Aids-Pille“) verhindert zwar die Ansteckung mit HIV mit sehr hoher Sicherheit, war aber anfangs aus verschiedenen Gründen umstritten [98]. Für die quantitativ manuelle Analyse nahm das Forschungsteam alle *N* = 217 englischsprachigen PrEP-Videos auf Youtube in ihr Sample auf, die zum Untersuchungszeitpunkt mehr als 100-mal abgerufen worden waren. Diese Videos wurden mit einem Codebuch codiert, dessen Kategorien sich auf Kernelemente der Darstellung bezogen (z. B. Informationen über den Zugang zur PrEP und die Kosten der PrEP).

Es waren auch 4 quantitative Studien zu finden (davon 1 zu Instagram und 3 zu Twitter), die sich auf eine computationale Methode der Medieninhaltsanalyse stützen, also das Social-Media-Material durch einen Computeralgorithmus analysieren. Im Sinne von Big-Data-Analysen sind dabei bislang Stichprobenumfänge im fünf- bis sechsstelligen Bereich verarbeitet worden; wesentlich größere Stichproben wären jedoch methodisch möglich. Eine quantitativ computationale Analyse wollte beispielsweise herausfinden, wie die HIV-PrEP auf Twitter diskutiert wird [47]. Dazu wurde eine Stichprobe von *N* = 624.569 englischsprachigen Twitter-Tweets mit PreP-Bezug aus den Jahren 2015 und 2016 gezogen und unter anderem mittels *Sentiment-Analyse* computerbasiert ausgewertet. Auf diese Weise sollten positive und negative PrEP-Tweets unterschieden und Hauptargumente pro und kontra PrEP auf Twitter identifiziert werden.

#### Methoden der Qualitätsanalyse

Von den 72 in das Forschungsreview eingeschlossenen Analysen von Gesundheitsinformationen in sozialen Medien enthalten 10 (13,9 %) eine systematische Qualitätsanalyse des Materials, wobei unterschiedliche selbstkonstruierte sowie etablierte Verfahren der Qualitätsmessung zum Einsatz kommen (F3). Zu den etablierten Verfahren gehören beispielsweise die Qualitätsmessung mit dem originalen oder modifizierten *DISCERN-Index* [99, 100] oder gemäß *Global Quality Score* (GQS; [101]). Der GQS ist ein Gesamtmaß für die Qualität von Online-Gesundheitsinformationen, das aus einer einzigen fünfstufigen Rankingskala besteht von 1 (geringe Qualität) bis 5 (exzellente Qualität). Der DISCERN-Index dagegen erfasst neben der Gesamtqualität auf 15 weiteren Items auch einzelne Qualitätsaspekte (z. B. wie gut die Informationen durch Quellen belegt sind, wie aktuell sie sind und wie ausgewogen sie präsentiert werden), wobei ebenfalls fünfstufige Ratingskalen zum Einsatz kommen. Diese Verfahren wurden z. B. eingesetzt, um die Qualität von YouTube-Videos einzuschätzen, die Informationen zu Beckenbodenübungen [51], zur Behandlung von vorzeitiger Ejakulation [25] oder zur Verhütung mit dem Hormonimplantat bieten [88]. In qualitativen Studien erfolgte zuweilen eine kursorische Qualitätsbewertung im Diskussionsteil [77, 90].

### Ergebnisse der bisherigen Forschung

#### Anbieter der sexuellen Gesundheitsinformationen

Sexuelle Gesundheitsinformationen in sozialen Medien werden laut bisherigem Forschungsstand mehrheitlich von Gesundheitslaien (86,1 % der Publikationen im Sample) angeboten (F4), gefolgt von Gesundheitsprofis (Organisationen und Einzelpersonen zusammen: 66,7 %) und Medienprofis (Organisationen und Einzelpersonen zusammen: 33,3 %). (Dabei ist zu beachten, dass manche Studien Social-Media-Informationen von mehreren Anbietern analysierten und sich deshalb die Prozentwerte nicht zu 100 % addieren.) Andere kommerzielle Unternehmen sind seltener vertreten (Tab. 2). Eine Analyse von *N* = 259 Instagram-Accounts zum Thema Unfruchtbarkeit zeigte beispielsweise, dass diese sowohl von Gesundheitslaien (z. B. Patient:innen, Betroffenen) als auch von Gesundheitsprofis (z. B. Ärzt:innen, Kliniken) betrieben werden, wobei die Laien aber wesentlich größere Reichweiten bis hin zum Influencer-Status (definiert über mehr als 10.000 Follower) erreichten [44]. Die bislang einzige Snapchat-Studie [72] untersuchte, welche sexuellen Gesundheitsinformationen über die Snapchat-Accounts von 2 Frauenzeitschriften (*Cosmopolitan, SELF*) und 2 Männermagazinen (*GQ, Esquire*) verbreitet werden, hier sind also Medienprofis (Zeitschriftenredaktionen) die Anbieter.

**Table 2:** 

Anbieter der sexuellen Gesundheitsinformationen	Anzahl der Studien^a^ (Anteile in %)	YouTube	Facebook	Instagram	Snapchat	Twitter
Laien/Peer Sex Educators	62 (86,1)	36 (50,0)	3 (4,2)	3 (4,2)	0 (0)	20 (27,8)
Organisationen der sexuellen Gesundheit	27 (37,5)	14 (19,4)	1 (1,4)	2 (2,8)	0 (0)	10 (13,9)
Medienorganisationen/Fachleute für Medien und Kommunikation	24 (33,3)	13 (18,1)	1 (1,4)	1 (1,4)	1 (1,4)	8 (11,1)
Fachleute für sexuelle Gesundheit	21 (29,2)	16 (22,2)	0 (0)	1 (1,4)	0 (0)	4 (5,6)
Kommerzielle Anbieter	17 (23,6)	10 (13,9)	1 (1,4)	1 (1,4)	0 (0)	5 (6,9)
Anderer Anbieter	1 (1,4)	0 (0)	0 (0)	0 (0)	0 (0)	1 (1,4)

^a^*N* = 72. Berichtet werden die absoluten und relativen Häufigkeiten der Publikationen. Da manche Studien Social-Media-Informationen von mehreren verschiedenen Anbietern analysierten und die Anbieterkategorien somit nicht exklusiv sind, addieren sich die Prozentwerte nicht zu 100 %

#### Zielgruppen der sexuellen Gesundheitsinformationen

Die bisherige Forschung zu sexuellen Gesundheitsinformationen in sozialen Medien gibt wenig Auskunft über die jeweils intendierten und erreichten Zielgruppen des Materials (F5): Mehrheitlich wird in den Studien weder berichtet, welche Geschlechter jeweils angesprochen werden (nicht berichtet in 65,3 % der untersuchten Publikationen) oder erreicht werden (nicht berichtet: 73,6 %), noch, welche sexuellen Identitäten angesprochen werden (nicht berichtet: 83,3 %) oder erreicht werden (nicht berichtet: 90,3 %). Auch zu angezielten und erreichten Altersgruppen sind in den Studien kaum Informationen zu finden.

#### Themen und Wissensformen der sexuellen Gesundheitsinformationen

Hinsichtlich der behandelten Themenbereiche sexueller Gesundheit zeigen die bisherigen Studien eine breite Streuung (F6; Tab. 3). Beispielsweise ist untersucht worden, wie die Impfung gegen humane Papillomviren (HPV) auf YouTube [48] und Twitter [79] dargestellt wird (Themengebiet HIV/STI), wie Trans*Personen auf YouTube ihre sexuelle Gesundheit beschreiben [91] und welche alternativen Männlichkeitsbilder auf YouTube [84] vermittelt werden (Themengebiet geschlechtliche und sexuelle Identitäten), wie Mädchen und Frauen sexuelle Gewalt auf YouTube [59] behandeln und welche Möglichkeiten der sexuellen Gewaltprävention auf Twitter [64] diskutiert werden (Themengebiet sexuelle Gewalt), welche Verhütungsinformationen auf YouTube [85] verfügbar sind (Themengebiet Fruchtbarkeit und Verhütung), welche Informationen zu erektiler Dysfunktion auf YouTube [57] oder zu Unfruchtbarkeit auf Instagram und Twitter [44] verbreitet werden (Themengebiet sexuelle und reproduktive Störungen) und welche Informationen über lesbische Lebensweisen [80] oder sexuelle Techniken [33] auf YouTube zu finden sind (Themengebiet sexuelle Funktionen, Lebensstile und Vergnügen).

**Table 3:** 

Themen	Anzahl der Studien^a^ (Anteile in %)	YouTube	Facebook	Instagram	Snapchat	Twitter
HIV/STI	24 (33,3)	9 (12,5)	3 (4,2)	2 (2,8)	1 (1,4)	9 (12,5)
Geschlechtliche und sexuelle Identitäten	24 (33,3)	19 (26,4)	2 (2,8)	1 (1,4)	0 (0)	2 (2,8)
Sexuelle Gewalt	22 (30,6)	5 (6,9)	2 (2,8)	2 (2,8)	1 (1,4)	12 (16,7)
Fruchtbarkeit und Verhütung	21 (29,2)	12 (16,7)	3 (4,2)	1 (1,4)	1 (1,4)	4 (5,6)
Sexuelle und reproduktive Störungen	12 (16,7)	9 (12,5)	2 (2,8)	0 (0)	1 (1,4)	0 (0)
Sexuelle Funktionen, Lebensstile und Vergnügen	12 (16,7)	7 (9,7)	3 (4,2)	0 (0)	1 (1,4)	1 (1,4)

^a^*N* = 72. Berichtet werden die absoluten und relativen Häufigkeiten der Publikationen. Da manche Studien Social-Media-Informationen mit mehreren verschiedenen Themen analysierten und die Themenkategorien somit nicht exklusiv sind, addieren sich die Prozentwerte nicht zu 100 %

Die sexuellen Gesundheitsinformationen in sozialen Medien, die von den vorliegenden Studien untersucht werden, behandeln die genannten Themen sehr viel häufiger rein auf der Basis von Erfahrungswissen (44,4 % der untersuchten Publikationen) oder einer Mischung von Erfahrungs- und Faktenwissen (29,2 %) als auf der Basis von reinem Faktenwissen (19,4 %). Bei einem kleinen Teil der Publikationen war die Art des vermittelten Wissens unklar (6,9 %). Eine Analyse von Instagram-Accounts zum Thema Unfruchtbarkeit zeigte beispielsweise, dass die vermittelten Informationen ganz überwiegend Erfahrungswissen darstellen in Form von persönlichen Erfahrungsberichten und gegenseitiger Ermutigung von Betroffenen, während Informationen auf der Basis von Faktenwissen (z. B. aktueller Forschungsstand über Methoden der Unfruchtbarkeitsbehandlung) viel weniger vertreten waren [44].

#### Qualität der sexuellen Gesundheitsinformationen

10 der eingeschlossenen Publikationen enthalten eine standardisierte Qualitätsanalyse der sexuellen Gesundheitsinformationen, wobei 8 dieser durchgängig aus der Medizin stammenden Studien globale Qualitätseinschätzungen des Materials liefern (Tab. 4). Insgesamt fallen die Qualitätsbewertungen eher negativ aus (F7): Berichtet werden Defizitraten für unterschiedliche Indikatoren zwischen 12 % und 93 %. Die Werte des fünfstufigen GQS und DISCERN-Index bewegen sich überwiegend im unteren bis mittleren Skalenbereich. Informationen von Gesundheitsprofis schneiden bei der Qualitätsbeurteilung regelmäßig besser ab als Informationen von Laien.

**Table 4:** 

Studien mit Messung der Informationsqualität	Untersuchungsgegenstand	Qualitätsbeurteilung
Culha et al. (2021; [51])	*N* = 59 englischsprachige YouTube-Videos zum Beckenbodentraining	88 % der Videos (52) nützlich („useful“) mit höherem DISCERN-Index (M = 3,14)
		**12** **% der Videos **(7) irreführend („misleading“) mit geringerem DISCERN-Index (M = 1,29)
Ekram et al. (2019; [55])	*N* = 35 englischsprachige YouTube-Videos zur HPV-Impfung	31 % der Videos (11) impfbefürwortend, was der Evidenzlage entspricht
		11 % der Videos (4) neutral
		**57** **% der Videos **(20) impfkritisch, was der Evidenzlage widerspricht
Fode et al. (2020; [57])	*N* = 92 englischsprachige YouTube-Videos mit medizinischen Informationen über erektile Dysfunktion	19 % der Videos (18) mit guter oder sehr guter Zuverlässigkeit (DISCERN-Skala 4–5)
		23 % der Videos (21) mit mittlerer Zuverlässigkeit (DISCERN-Skala 3)
		**58** **% der Videos** (53) mit schlechter oder sehr schlechter Zuverlässigkeit (DISCERN-Skala 1–2)
Gul und Diri (2019; [25])	*N* = 132 englischsprachige YouTube-Videos zur Behandlung von vorzeitiger Ejakulation	70 % der Videos (93) zuverlässig („reliable“) mit höherem GQS (M = 2,74)
		**30** **% der Videos **(39) unzuverlässig („unreliable“) mit geringerem GQS (M = 1,15)
Hansen et al. (2016; [63])	*N* = 314 englischsprachige YouTube-Videos zur Medikamenteneinnahme in der Schwangerschaft mit Fokus auf teratogene bzw. reproduktionstoxische Effekte	Keine Gesamtbewertung der Qualität der Informationen in der Stichprobe vorgenommen
Khawaja et al. (2017; [70])	*N* = 291 Organisationen der sexuellen und reproduktiven Gesundheit und deren Facebook-Aktivitäten in konservativen asiatischen Ländern	**89** **% der Organisationen** verfügen *nicht *über eine landesspezifische Facebook-Seite mit mindestens geringer Rate of Activity (ROA) definiert als mind. 1 Beitrag pro Monat
		**93** **% der Organisationen** verfügen *nicht* über eine landesspezifische Facebook-Seite mit mindestens geringer Rate of User Activity (RUA) definiert als mind. 8 User-Interaktionen pro Monat
		**93** **% der Organisationen** verfügen *nicht* über eine Facebook-Seite mit mindestens geringer Reichweite definiert als mind. 500 Follower/Likes
Ku et al. (2020; [71])	*N* = 42 reichweitenstärkste, englischsprachige YouTube-Videos zu männlicher Unfruchtbarkeit	10 % der Videos (4) mit guter oder sehr guter Schulnote laut Expertenurteil
		**90** **% der Videos **(38) mit mittlerer oder schlechter Schulnote laut Expertenurteil
Paul et al. (2017; [88])	*N* = 50 meistgesehene englischsprachige YouTube-Videos zum Hormonimplantat als Verhütungsmethode	24 % der Videos (12) von Gesundheitsprofis mit höherem DISCERN-Index (Md = 3)
		**76** **% der Videos** (38) von Patient:innen mit geringerem DISCERN-Index (Md = 2)
Warren et al. (2021; [96])	*N* = 72 meistgesehene englischsprachige YouTube-Videos zu männlichem Hypogonadismus und Testosteronersatztherapie	38 % der Videos (27) von Gesundheitsprofis mit höherem DISCERN-Index (M = 3,22)
		**62** **% der Videos **(45) von Gesundheitslaien mit geringerem DISCERN-Index (M = 1,87)
Whiteley et al. (2020; [97])	*N* = 58 englischsprachige YouTube-Videos zur HIV-Präexpositionsprophylaxe (HIV-PrEP)	Keine Gesamtbewertung der Qualität der Informationen in der Stichprobe vorgenommen

*n* = 10 Publikationen mit standardisierter Messung der Informationsqualität. Berichtet werden ausgewählte prozentuierte Qualitätsindikatoren, wobei absolute Häufigkeiten in Klammern angegeben sind

Der DISCERN-Index und der Global Quality Score (GQS) haben jeweils einen Wertebereich von 1 (geringe Qualität) bis 5 (exzellente Qualität)

Die Prozentwerte für Indikatoren schlechter Qualität sind fett gedruckt

*M* Mittelwert, *Md* Median

Bei kursorischen Qualitätseinschätzungen in qualitativen Studien wird meist kritisiert, dass die untersuchten sexuellen Gesundheitsinformationen nicht divers genug seien und somit letztlich zu enge normative Vorstellungen vermittelten, etwa wenn es um selbstbestimmte Hausgeburt [77] oder geschlechtliche Transition [90] geht.

## Diskussion

### Interpretation der Befunde zu den 7 Forschungsfragen

Die bisherigen Forschungsaktivitäten verteilen sich sehr ungleich auf die verschiedenen Social-Media-Plattformen (F1). Starke Forschungsaktivität ist zu verzeichnen bei Plattformen mit hoher Reichweite (YouTube) und bei Plattformen mit guten Möglichkeiten der Datengewinnung über eine öffentliche Plattform-API (Application Programming Interface), die z. B. den automatischen Download von Beiträgen und Metadaten erlaubt (YouTube, Twitter). Insbesondere zu neueren Plattformen (z. B. TikTok) waren trotz großer Reichweite und nennenswerter Verbreitung sexueller Gesundheitsinformationen [9] zum Erhebungszeitpunkt keine empirischen Studien auffindbar.

Die bisherige Forschung nutzt alle 3 verfügbaren methodischen Ansätze der empirischen Medieninhaltsanalyse (qualitativ, quantitativ manuell und quantitativ computational), wobei computionale Analysen noch relativ selten sind (F2). Jenseits des Fokus dieses Reviews liegen zudem nichtempirische Studien mit geistes- und kulturwissenschaftlichem Hintergrund vor, die sich sexuellen Gesundheitsinformationen in sozialen Medien interpretativ nähern, ohne dass jedoch ausreichende methodische Details zur Stichprobenauswahl und Datenanalyse angegeben sind, um die Studie nachvollziehbar und replizierbar zu machen (z. B. [102]).

Angesichts anhaltender Debatten über die Qualität von Online-Informationen im Allgemeinen und von sexuellen Gesundheitsinformationen im Besonderen [9, 10] ist bedauernd zu konstatieren, dass Qualitätsanalysen bislang im Forschungsfeld rar sind (F3). Wenn sie vorgenommen werden, liegt der Fokus bisher eher auf nichtintendierten Lücken und Fehlern („misinformation“), kaum auf gezielter Falschinformation im Sinne von bewusster Täuschung und Irreführung („disinformation“, „fake news“). Dabei gibt es eine Reihe von Themen der sexuellen und reproduktiven Gesundheit (z. B. Homosexualität, Transition, sexuelle Gewalt, Schwangerschaftsabbruch, Verhütung), zu denen bewusst ideologisch und/oder kommerziell motivierte Falschinformationen in sozialen Medien verbreitet werden. Nicht nur Desinformation ist bislang im Kontext von Online-Gesundheitsinformationen zu wenig untersucht, auch Qualitätsmängel bei den Nutzerkommentaren sollten stärker beachtet werden, da Nutzerkommentare z. B. Hassbotschaften oder Verschwörungsmythen enthalten können [55, 103].

Das Spektrum der Anbieter:innen sexueller Gesundheitsinformationen in sozialen Medien ist breit und umfasst neben privaten Einzelpersonen, die wegen fehlender formaler Qualifikation als Gesundheitslaien klassifiziert werden, vor allem Gesundheitsprofis sowie Medienprofis. Bei Gesundheits- und Medienprofis können sowohl Organisationen (z. B. Kliniken, Zeitschriften) als auch Einzelpersonen (z. B. Ärzt:innen, Journalist:innen) als Content-Produzent:innen und Betreiber:innen von Social-Media-Accounts sichtbar werden (F4). Insgesamt sind in den bisherigen Studien zu sexuellen Gesundheitsinformationen in sozialen Medien die Gesundheitslaien am stärksten vertreten. Und es wird auch mehrfach darauf verwiesen, dass diese Anbietergruppe die meisten und reichweitenstärksten Beiträge liefern. Dass die Stimmen von Patient:innen bzw. Betroffenen und Beteiligten in sozialen Medien so viel Raum einnehmen, lässt sich als Beitrag zu mehr Partizipation an der öffentlichen Gesundheitskommunikation werten und würdigen. Gleichzeitig verweisen diese Befunde auch auf einen gewissen Nachholbedarf bei Gesundheitsprofis, sich sichtbarer an der sexualbezogenen Gesundheitskommunikation auf führenden Social-Media-Plattformen zu beteiligen.

Während nicht-öffentliche Online-Interventionen stets mit klaren und oft recht engen Zielgruppendefinitionen arbeiten, ist im Feld der öffentlichen Online-Informationen zur sexuellen Gesundheit das Publikum breiter bzw. weniger spezifiziert (F5). So spricht beispielsweise das lesbische Coming-out-Video einer bekannten deutschsprachigen Influencerin auf YouTube („Coming Out | Melina Sophie“) nicht nur die lesbische Zielgruppe an, sondern zieht deutlich größere Kreise (6 Mio. Abrufe). Es ist eine weitgehend offene Forschungsfrage, welche Zielgruppen mit konkreten sexuellen Gesundheitsinformationen in sozialen Medien jeweils erreicht werden.

Bei der Interpretation der Daten zur Repräsentation verschiedener Themen der Sexualaufklärung (F6) muss man sich vor Augen führen, dass die Studienergebnisse kein Spiegel der Social-Media-Welt sind, sondern lediglich ein Spiegel der Themenauswahl der Forschenden. Da ein Teil der Forschung zu sexuellen Gesundheitsinformationen in sozialen Medien aus der Medizin stammt, ist es z. B. naheliegend, dass man sich in diesen Studien auf die klassischen medizinischen Themen der Sexualaufklärung fokussiert (HIV/STI, sexuelle und reproduktive Störungen, Fruchtbarkeit und Verhütung). Ein anderer Teil der Studien stammt aus den Medien- und Sozialwissenschaften, hier liegt der Fokus dann stärker auf Fragen der medialen Sichtbarkeit von sexueller Vielfalt (z. B. geschlechtliche und sexuelle Identitäten) oder auf sexualpolitischen Diskursen (z. B. gesellschaftlicher Umgang mit sexueller Gewalt und entsprechender Online-Aktivismus). Auffällig ist, dass die bisherige Forschung die vielfältigen Social-Media-Inhalte zu sexuellem Wohlbefinden und Vergnügen noch nicht sehr umfassend aufgegriffen hat [10]. Dass die in sozialen Medien verbreiteten sexuellen Gesundheitsinformationen zu verschiedenen Themen laut bisheriger Forschung mehrheitlich auf Erfahrungswissen basieren, ist insofern schlüssig, als eben Gesundheitslaien als Kommunikator:innen überwiegen. Der Austausch von Erfahrungen aus erster Hand kann hilfreich sein, positive Rollenmodelle bieten und zum Empowerment (Autonomie und Selbstbestimmung) beitragen. Gleichzeitig birgt der starke Fokus auf Erfahrungswissen gegenüber wissenschaftlich gesichertem Faktenwissen die Gefahr, dass subjektive Einzelerfahrungen in ihrer Verallgemeinerbarkeit überschätzt werden.

Obwohl die Sorge über mangelnde Qualität sexueller Gesundheitsinformationen in sozialen Medien weit verbreitet ist, muss man die bisherige Datenlage als dürftig bezeichnen. Dabei scheinen die wenigen Daten die Sorgen über Qualitätsmängel zu bestätigen (F7). Wichtig bei der Qualitätsbetrachtung ist jedoch die Frage nach den relevanten Standards. Von Online-Gesundheitsinformationen eine perfekte Qualität zu erwarten scheint überzogen. Notwendig zur Einschätzung wären daher realistische Vergleichswerte [9, 33]: Welche Qualität haben sexuelle Gesundheitsinformationen, die offline verbreitet werden, etwa über Printbroschüren oder mündliche Botschaften im Zuge der Sexualaufklärung in Schulen, Familien oder Arztpraxen? Derartige Vergleichsstudien fehlen völlig.

### Limitationen

Obwohl zur Identifikation der Literatur für das vorliegende Scoping Review bewusst eine breite Suchstrategie genutzt wurde, erwies es sich als schwierig, das gesamte Spektrum möglicher Themen der sexuellen und reproduktiven Gesundheit erschöpfend abzubilden. Manche Publikationen zum Thema enthalten das Stichwort „sexuell“/„sexual“ nicht und wurden vermutlich auch durch die ergänzende manuelle Suche über die Literaturverzeichnisse und Google Scholar nicht aufgefunden. Eine weitere Limitation bezieht sich auf das Spektrum der berücksichtigten Social-Media-Plattformen, das aus forschungsökonomischen Gründen auf die 8 in Deutschland populärsten begrenzt wurde. Nicht zuletzt ist die Generalisierbarkeit des vorliegenden Scoping Reviews dadurch begrenzt, dass nur englisch- und deutschsprachige Literatur berücksichtigt werden konnte.

### Ausblick

Der durch das vorliegende Scoping Review vermittelte Gesamtüberblick und die selektiven Einblicke in einzelne Studien mögen dazu anregen, die sexuellen Gesundheitsinformationen in sozialen Medien in Zukunft verstärkt zu untersuchen. Nur wenn wir genauer wissen, wer auf welchen Social-Media-Plattformen welche sexuellen Gesundheitsinformationen verbreitet, können wir die diesbezügliche Gesundheitskompetenz der Zielgruppen fördern, indem wir sie bei der Auswahl und Einordnung dieser Online-Informationen unterstützen. So könnte beispielsweise die schulische Sexualaufklärung populäre YouTube-Videos zu Fragen der sexuellen Gesundheit (z. B. Coming-out, Verhütung, sexuelle Gewalt, Selbstbefriedigung) aufgreifen und deren Botschaften sowie die zugehörigen Diskussionen in der Kommentarspalte mit Blick auf Informationsnutzen und Informationsqualität kritisch analysieren. Ebenso könnten professionelle Aufklärungsmaterialien und Patienteninformationen (Broschüren, Flyer, Websites) darauf eingehen, welche Thementrends und Accounts in sozialen Medien den Diskurs über sexuelle und reproduktive Gesundheit mitbestimmen, diesbezügliche Empfehlungen oder Warnungen aussprechen und häufige Fragen aufgreifen. Nicht zuletzt bleibt es eine Aufgabe für die professionellen Akteur:innen im Feld der sexuellen und reproduktiven Gesundheit, mit ihren Informationen auch selbst auf Social-Media-Plattformen sichtbarer zu werden.

## Supplementary Information




