# Supplementary material for: Sexual health information on social media: a systematic scoping review
Source: Bundesgesundheitsblatt Gesundheitsforschung Gesundheitsschutz. 2021 Oct 14;64(11):1416–29. [Article in German] doi: 10.1007/s00103-021-03431-9 (PMC8550659; doi:10.1007/s00103-021-03431-9)
Supplement: Supplementary file 1 [file 103_2021_3431_MOESM1_ESM.pdf]

Bundesgesundheitsblatt 11/2021

Onlinematerial zum Beitrag:

Sexuelle Gesundheitsinformationen in sozialen Medien: Ein systematisches Scoping Review

Sexual Health Information on Social Media: A Systematic Scoping Review

Nicola Döring[1], Melisa Conde[1]

[1]Technische Universität Ilmenau, Institut für Medien und Kommunikationswissenschaft, Ilmenau, Deutschland

Korrespondenzadresse

Prof. Dr. Nicola Döring
TU Ilmenau, IfMK
Ehrenbergstraße 29
98693 Ilmenau
Deutschland
nicola.doering@tu-ilmenau.de

Inhalte:

A. Search Syntax for Databases (conducted on 10.02.2021)

B. Codebook

C. Intercoder Reliability of Codebook

Anmerkung:

Da hauptsächlich englischsprachige Fachliteratur in das Scoping Review eingeflossen ist, sind die Begleitmaterialien englischsprachig. Der Originaldatensatz ist ergänzend auf https://osf.io/c8jbx/ abgelegt.

A. Search Syntax for Databases (conducted on 10.02.2021)

1. **MEDLINE**

1.1 (TS = YouTube OR TI = YouTube) AND (TS = (sexual* OR sexuell*) OR (TI = (sexual* OR sexuell*)))
→ n = 32

1.2 (TS = Facebook OR TI = Facebook) AND (TS = (sexual* OR sexuell*) OR (TI = (sexual* OR sexuell*)))
→ n = 303

1.3 (TS = Instagram OR TI = Instagram) AND (TS = (sexual* OR sexuell*) OR (TI = (sexual* OR sexuell*)))
→n = 32

1.4 (TS = Snapchat OR TI = Snapchat) AND (TS = (sexual* OR sexuell*) OR (TI = (sexual* OR sexuell*)))
→n = 8

1.5 (TS = Twitter OR TI = Twitter) AND (TS = (sexual* OR sexuell*) OR (TI = (sexual* OR sexuell*)))
→n = 86

1.6 (TS = TikTok OR TI = TikTok) AND (TS = (sexual* OR sexuell*) OR (TI = (sexual* OR sexuell*)))
→n = 1

1.7 (TS = Twitch OR TI = Twitch) AND (TS = (sexual* OR sexuell*) OR (TI = (sexual* OR sexuell*)))
→n = 68

1.8 (TS = Pinterest OR TI = Pinterest) AND (TS = (sexual* OR sexuell*) OR (TI = (sexual* OR sexuell*)))
→n = 2

2. **PsycINFO**

2.1 (TI = YouTube OR AB = YouTube) AND (TI = (sexual* OR sexuell*) OR AB = (sexual* OR sexuell*))
→n = 44

2.2 (TI = Facebook OR AB = Facebook) AND (TI = (sexual* OR sexuell*) OR AB = (sexual* OR sexuell*))
→n = 265

2.3 (TI = Instagram OR AB = Instagram) AND (TI = (sexual* OR sexuell*) OR AB = (sexual* OR sexuell*))
→n = 34

2.4 (TI = Snapchat OR AB = Snapchat) AND (TI = (sexual* OR sexuell*) OR AB = (sexual* OR sexuell*))
→n = 13

2.5 (TI = Twitter OR AB = Twitter) AND (TI = (sexual* OR sexuell*) OR AB = (sexual* OR sexuell*))
→n = 75

2.6 (TI = TikTok OR AB = TikTok) AND (TI = (sexual* OR sexuell*) OR AB = (sexual* OR sexuell*))
→n = 0

2.7 (TI = Twitch OR AB = Twitch) AND (TI = (sexual* OR sexuell*) OR AB = (sexual* OR sexuell*))
→n = 5

2.8 (TI = Pinterest OR AB = Pinterest) AND (TI = (sexual* OR sexuell*) OR AB = (sexual* OR sexuell*))
→n = 2

3. **Scopus**

3.1 (TITLE-ABS-KEY(YouTube AND (sexual* OR sexuell*)))
→n = 154

3.2 (TITLE-ABS-KEY(Facebook AND (sexual* OR sexuell*)))
→n = 581

3.3 (TITLE-ABS-KEY(Instagram AND (sexual* OR sexuell*)))
→n = 102

3.4 (TITLE-ABS-KEY(Snapchat AND (sexual* OR sexuell*)))
→n = 26

3.5 (TITLE-ABS-KEY(Twitter AND sexual* OR sexuell*))
→n = 268

3.6 (TITLE-ABS-KEY(TikTok AND sexual* OR sexuell*))
→n = 3

3.7 (TITLE-ABS-KEY(Twitch AND sexual* OR sexuell*))
→n = 141

3.8 (TITLE-ABS-KEY(Pinterest AND sexual* OR sexuell*))
→n = 4

B. Codebook

Inclusion criteria:

1) Study addresses **sexual health information** on one or more of **eight** selected social media platforms (1. YouTube, 2. Facebook, 3. Instagram, 4. Snapchat, 5. Twitter, 6. TikTok, 7. Twitch, and/or 8. Pinterest). Only sexual information that creates/develops **knowledge, attitudes, and skills** to protect and advance a person's **sexual health, rights, and well-being** should be included.

2) Study examines the **content and/or quality** of this health information.

3) Study is **empirically oriented**, i.e., data are evaluated, the collection and analysis of which are presented in a methodologically comprehensible manner.

4) Publication is in **German** or **English** language.

5) Publication with scientific quality assurance (**peer review process**).

Study characteristics

Item	Coding values		Description
1. Citation	-	Open coding	Author(s), title, publication outlet
2. Publication date	-	Open coding	Year of publication
3. Country of study	-	Open coding	Name of country
4. Language	1	English	Language in which study was published
	2	German	
5. Academic discipline	-	Open coding	Academic discipline of authors
6. Google citations	-	Open coding	Number of citations in Google Scholar

RQ1: How many studies are available on sexual health information on the eight selected social media platforms (1. YouTube, 2. Facebook, 3. Instagram, 4. Snapchat, 5. Twitter, 6. TikTok, 7. Twitch, and 8. Pinterest)?

Item	Coding values		Description
7. YouTube	0	No	The study examined sexual information on YouTube (exclusively or in addition to another platform and/or topic)
	1	Yes	
8. Facebook	0	No	The study examined sexual information on Facebook (exclusively or in addition to another platform and/or topic)
	1	Yes	
9. Instagram	0	No	The study examined sexual information on Instagram (exclusively or in addition to another platform and/or topic)
	1	Yes	
10. Snapchat	0	No	The study examined sexual information on Snapchat (exclusively or in addition to another platform and/or topic)
	1	Yes	
11. Twitter	0	No	The study examined sexual information on Twitter (exclusively or in addition to another platform and/or topic)
	1	Yes	
12. TikTok	0	No	The study examined sexual information on TikTok (exclusively or in addition to another platform and/or topic)
	1	Yes	
13. Twitch	0	No	The study examined sexual information on Twitch (exclusively or in addition to another platform and/or topic)
	1	Yes	
14. Pinterest	0	No	The study examined sexual information on Pinterest (exclusively or in addition to another platform and/or topic)
	1	Yes	

Note. Code only 1 platform with “yes”. Studies analyzing more than one platform must be charted separately for each platform.

RQ2: Which methods for analyzing the content of sexual health information in social media have been used in research to date?

Item	Coding values		Description
15. Qualitative content analysis	0<br>1	No<br>Yes	The method used for data collection was qualitative media content analysis of sexual education information
16. Quantitative manual content analysis	0<br>1	No<br>Yes	The method used for data collection was quantitative manual media content analysis of sexual education information
17. Quantitative computational content analysis	0<br>1	No<br>Yes	The method used for data collection was quantitative automated / computational media content analysis of sexual education information
18. Description of content analysis method	-	Open coding	Description of the specific method applied for content analysis (type of coding, software, methodology, etc.), when available
19. Total sample size	-	Open coding	Total sample size of study (including all analyzed platforms)
20. Unit of analysis for total sample	-	Open coding	Videos, channels, posts, tweets, themes, words, images, etc.
21. Platform sample size	-	Open coding	Sample size for specific platform (Number of YouTube videos, number of Facebook posts, etc.)
22. Unit of analysis for platform sample	-	Open coding	Videos, channels, posts, tweets, themes, words, images, etc.

RQ3: Which methods for assessing the quality of sexual health information in social media have been used in research to date?

Item	Coding values		Description
23. Quality assessment	0<br>1	No<br>Yes	Was the quality of the analyzed information assessed?
24. Assessment methods	-	Open coding	If quality was assessed, which method was used (scale, framework, etc.)

RQ4: From whom did the sexual health information studied on social media originate?

Item	Coding values		Description
25. Sexual health organizations	0<br>1	No<br>Yes	Leading institutions of sexual health promotion and sex education such as Planned Parenthood, the Centers for Disease Control and Prevention (CDC), the WHO, Universities or research institutes were a source of information
26. Sexual health professionals	0<br>1	No<br>Yes	Individual professional sex educators (individual physicians, scientists, sex therapists, etc.) were a source of information.
27. Media organizations and communications professionals	0<br>1	No<br>Yes	TV stations, newspapers, publishers, etc. and/or individual journalists, authors, etc. were a source of information (NY Times, Vogue, CNN, Buzzfeed, etc.)
28. Lay people/peer sex educators	0<br>1	No<br>Yes	Individuals with no formal training in media and/or sexual health (who speak primarily from own experience) were a source of information
29. Corporate	0<br>1	No<br>Yes	A company, brand, etc. (condom company, pharmaceutical company, sex-toy store, etc.) were a source of information
30. Other	-	Open coding	Source of information not included in previous categories (mention category)
31. Further source description	-	Open coding	Additional qualitative description of source of information

RQ5: What are the target audiences for the sexual health information studied on social media?

Item		Coding values	Description
32. Gender (intended target)	0	Not mentioned	Study does not mention gender of intended target group
	1	Female	Cisgender (mentioned or implied)
	2	Male	Cisgender (mentioned or implied)
	3	Transgender	Both female and male identified and trans* identified
	4	Intersex/intergender	Intersex/intergender
	5	Other	If the audience's gender is not included in the above categories or if more than one category is mentioned, code 5 and chart in "further description" with qualitative information
33. Further gender description (intended target)	-	Open coding	Code any additional gender here (non-binary, gender-queer, gender-diverse, etc.) or any combination of categories (i.e., female and male audience, etc.)
34. Age (intended target)	-	Open coding	Age (or age group) of those for which the sexual health information is intended (teenagers, young couples, older people, etc.)
35. Sexual identity (intended target)	0	Not mentioned	Study does not mention sexual identity of intended target group
	1	Heterosexual	Heterosexual
	2	Homosexual	Homosexual
	3	Bisexual	Bisexual
	4	Asexual	Asexual
	5	Other	If the audience's sexual identity is not included in the above categories or if more than one category is mentioned, code 5 and chart in "further description" with qualitative information
36. Further sexual identity description	-	Open coding	Code any additional sexual identity here (pansexual, demisexual, etc.) or any combination of categories (i.e., LGBTQ+ community in general, etc.)

37. Gender (reached target)	0	Not mentioned	Study does not mention gender of reached target group
	1	Female	Cisgender (mentioned or implied)
	2	Male	Cisgender (mentioned or implied)
	3	Transgender	Both female and male identified and trans* identified
	4	Intersex/intergender	Intersex/intergender
	5	Other	If the audience's gender is not included in the above categories or if more than one category is mentioned, code 5 and chart in "further description" with qualitative information
38. Further gender description	-	Open coding	Code any additional gender here (non-binary, gender-queer, gender-diverse, etc.) or any combination of categories (i.e., female and male audience, etc.)
39. Age (reached target)	-	Open coding	Age (or age group) of those for which the sexual health information is intended (teenagers, young couples, older people, etc.)
40. Sexual identity (reached target)	0	Not mentioned	Study does not mention sexual identity of reached target group
	1	Heterosexual	Heterosexual
	2	Homosexual	Homosexual
	3	Bisexual	Bisexual
	4	Asexual	Asexual
	5	Other	If the audience's sexual identity is not included in the above categories or if more than one category is mentioned, code 5 and chart in "further description" with qualitative information
41. Further sexual identity description	-	Open coding	Code any additional sexual identity here (pansexual, demisexual, etc.) or any combination of categories (i.e., LGBTQ+ community in general, etc.)
42. Additional audience characteristics	-	Open coding	Any additional characteristics that could be relevant for answering the RQs (Education, religious affiliation, etc.)

RQ6: What sexual and reproductive health topics does the sexual health information studied on social media address and what type of knowledge is provided?

Item	Coding values		Description
43. HIV/STI	0<br>1	No<br>Yes	The topic of HIV and/or sexually transmitted infections (including symptoms, treatment, etc.) was covered in the analyzed sexual health information (i.e., HIV, HPV, syphilis, gonorrhoea, chlamydia, etc.)
44. Fertility and contraception	0<br>1	No<br>Yes	The topics of fertility and contraception (planned or unplanned pregnancy, birth control methods, giving birth, etc.) were covered in the analyzed sexual health information.
45. Sexual violence	0<br>1	No<br>Yes	The topic of sexual violence was covered in the analyzed sexual health information (#metoo movement, support groups, disclosure of abuse stories, etc.)
46. Sexual and reproductive disorders	0<br>1	No<br>Yes	The topic of sexual and reproductive disorders was covered in the analyzed sexual health information (erectile disfunction, premature ejaculation, etc.)
47. Sexual and gender identities and diversity	0<br>1	No<br>Yes	The topic of sexual and gender identities and diversity was covered in the analyzed sexual health information (coming out experiences, LGBTQ+ support groups, transitioning, gender roles, etc.)
48. Sexual functions, lifestyle and pleasure	0<br>1	No<br>Yes	The topic of sexual functions, lifestyle and pleasure was covered in the analyzed sexual health information (sex toys, positive sexuality, etc.)
49. Further descriptions	-	Open coding	Additional information regarding included themes
50. Type of knowledge related to sexual health information	1<br>2<br>3	Factual<br>Factual and experiential<br>Experiential	Mainly or exclusively sharing factual knowledge<br>Partly sharing factual knowledge and partly sharing experiential knowledge<br>Mainly or exclusively sharing experiential knowledge
51. Additional information	-	Open coding	Further qualitative description of the type of knowledge shared

RQ7: What is the quality of the sexual health information studied on social media?

Item	Coding values		Description
52. Quality rating*	1	Good	Good / rather good information quality<br>0-10 % of research units show measured quality deficits
	2	Moderate	Moderate / partly good – partly poor information quality<br>11-50% of research units show measured quality deficits
	3	Poor	Rather poor / poor information quality<br>51-100% of research units show measured quality deficits

Note. *Rating as defined by author/s of original studies.

C. Intercoder Reliability of Codebook

Codebook Items	Cohen's Kappa	Agreement (%)
RQ1: Studies per platform		
YouTube	1	100
Facebook	1	100
Instagram	1	100
Snapchat	1	100
Twitter	1	100
TikTok	--[1]	100
Twitch	--[1]	100
Pinterest	--[1]	100
RQ2: Methods for analyzing content		
Qualitative content analysis		
Quantitative manual content analysis	.84	92
Quantitative computational content analysis	.90	95
	1	100
RQ3: Methods for assessing quality		
Quality assessment	.89	97
RQ4: Producers of sexual health information		
Sexual health organizations	.77	90
Sexual health professionals	.65	87
Media organizations / communications professionals	.83	92
Lay people/peer sex educators	.83	95
Corporate	.79	92
RQ5: Target audiences		
Gender (intended target) [a]	.80	92
Sexual identity (intended target) [b]	.89	97
Gender (reached target) [a]	1	100
Sexual identity (reached target) [b]	1	100
RQ6: Sexual and reproductive health topics and type of knowledge		
HIV/STI	.84	92
Fertility and contraception	.81	92
Sexual violence	.94	97
Sexual and reproductive disorders	.77	95
Sexual and gender identities and diversity	.78	90
Sexual functions, lifestyle and pleasure	.92	97
Type of knowledge [c]	.85	90
RQ7: Quality rating		
Quality rating [d]	.87	97
Total	.88	96

Note. Inter-coder reliability calculated for two coders. *N* = 38. All items coded as: 0. No / 1. Yes. [a] Gender coded as: 0. Not mentioned / 1. Female / 2. Male / 3. Transgender / 4. Intersex/intergender / 5. Other; [b] Sexual identity coded as: 0. Not mentioned / 1. Heterosexual / 2. Homosexual / 3. Bisexual / 4. Asexual / 5. Other; [c] Type of knowledge coded as: 1. Factual / 2. Factual and experiential / 3. Experiential. [d] Quality rating coded as: 1. Good / 2. Moderate / 3. Poor. --[1]: Reliability coefficient could not be computed.
